# Adoption, reach, and implementation of a cancer education intervention in African American churches

**DOI:** 10.1186/s13012-017-0566-z

**Published:** 2017-03-14

**Authors:** Sherie Lou Zara Santos, Erin K. Tagai, Mary Ann Scheirer, Janice Bowie, Muhiuddin Haider, Jimmie Slade, Min Qi Wang, Cheryl L. Holt

**Affiliations:** 1 Department of Behavioral and Community Health, University of Maryland, School of Public Health, 4200 Valley Dr., 1101 E SPH Building 255, College Park, MD 20742 USA; 2 Scheirer Consulting, Princeton, NJ USA; 3 Department of Health, Behavior and Society, Johns Hopkins Bloomberg School of Public Health, Baltimore, MD USA; 4 Institute for Applied Environmental Health, University of Maryland, School of Public Health, College Park, MD USA; 5 Community Ministry of Prince George’s County, Upper Marlboro, MD USA

## Abstract

**Background:**

Use of technology is increasing in health promotion and has continued growth potential in intervention research. Guided by the Reach, Effectiveness, Adoption, Implementation, ﻿and Maintenance (RE-AIM) framework, this paper reports on the *adoption*, *reach*, and *implementation* of Project HEAL (*Health through Early Awareness and Learning*)—a community-based implementation trial of a cancer educational intervention in 14 African American churches. We compare adoption, reach, and implementation at the organizational and participant level for churches in which lay peer community health advisors (CHAs) were trained using traditional classroom didactic methods compared with a new online system.

**Methods:**

Fifteen churches were randomized to one of two study groups in which two CHAs per church were trained through either classroom (“Traditional”; *n* = 16 CHAs in 8 churches) or web-based (“Technology”; *n* = 14 CHAs in 7 churches) training methods. Once trained and certified, all CHAs conducted a series of three group educational workshops in their churches on cancer early detection (breast, prostate, and colorectal). Adoption, reach, and implementation were assessed using multiple data sources including church-level data, participant engagement in the workshops, and study staff observations of CHA performance.

**Results:**

The project had a 41% overall *adoption* rate at the church level. In terms of *reach*, a total of 375 participants enrolled in Project HEAL—226 participants in the Traditional group (43% reach) and 149 in the Technology group (21% reach; *p* < .10). *Implementation* was evaluated in terms of adherence, dosage, and quality. All churches fully completed the three workshops; however, the Traditional churches took somewhat longer (*M* = 84 days) to complete the workshop series than churches in the Technology group (*M* = 64 days). Other implementation outcomes were comparable between both the Traditional and Technology groups (*p* > .05).

**Conclusions:**

Overall, the Project HEAL intervention had reasonable adoption, though reach could have been better. Implementation was strong across both study groups, suggesting the promise of using web-based methods to disseminate and implement evidence-based interventions in faith-based settings and other areas where community health educators work to eliminate health disparities.

## Background

It is well documented that a significant gap exists between research and practice [1]. Implementation research aims to close this gap by identifying and evaluating the processes necessary to ensure successful widespread translation of evidence-based interventions (EBIs) into real world settings [2–4]. Distinguishing implementation effectiveness from intervention effectiveness is critical for transporting interventions from the research setting to the real world [3]. When efforts to implement an intervention in a new setting fail, it is important to know if the failure occurred because the intervention was ineffective in the new setting (intervention failure) or if a good intervention was deployed incorrectly or weakly (implementation failure). Utilizing the the Reach, Effectiveness, Adoption, Implementation, and Maintenance (RE-AIM) framework [1, 5, 6] to evaluate implementation outcomes can be an effective approach to delineating between intervention and implementation failure.

### Role of technology in dissemination/implementation

Taking advantage of the technological advancements that are growing in popularity and accessibility can also be a useful tool in closing the gap between research discovery and program delivery [7, 8]. The use of interactive technologies (e.g., the Internet, social media, personal digital assistants, cellular phones, computer kiosks) have grown as platforms for health information dissemination, health-related behavior change, and decision-making for many age groups [9–12]. The “Health Online 2013” report by the Pew Foundation found that of the 81% of US adults who use the Internet, 59% report using it to obtain health information [10]. To date, effective e-health interventions have been documented over a wide array of health topics including, but not limited to, smoking cessation, weight management, anxiety and depression, and asthma management [11].

### Lay community health advisors

Peer educators or community health advisors (CHAs), in particular, are viewed as a promising strategy for successful health promotion program implementation [13]. CHAs have demonstrated their effectiveness in promoting health among groups in underserved communities that lack access to adequate care and health knowledge [14–16]. Ethnically, linguistically, socio-economically, and experientially indigenous to the communities in which they work, these trusted “insiders” serve as cost-effective resources and services to medically underserved populations [14, 17–19]. CHAs have been used to address a broad range of health issues [15, 20], and a number of studies have illustrated the ability of CHAs to do effective prevention work, reduce cultural and linguistic barriers to care, help patients successfully navigate complex health systems, and improve the quality and cost-effectiveness of care [14]. While the CHA approach has been found to be effective in addressing health disparities in many areas of chronic disease, training and technical assistance for CHAs requires considerable resources, in particular with regards to staffing.

### The present study—Project HEAL

Project HEAL (*Health through Early Awareness and Learning*) is a community-based cluster randomized implementation trial conducted in African American churches in Prince George’s County, MD, USA. Project HEAL is based on a foundation of three previous efficacy trials that established an evidence base for the breast, prostate, and colorectal cancer educational aspects of the intervention, respectively [21–23]. The previous colorectal and prostate efficacy trials utilized the same module-based CHA training approach that served as the basis for the Traditional approach used in the current Project HEAL. The previous trials used spiritually-based print educational materials culturally targeted for African Americans, which were distributed in group workshops in the churches. Combining all three interventions into one cohesive package [24], Project HEAL compared traditional classroom didactic methods with a new web-based system for training lay peer CHAs. To our knowledge, the current project is one of the first evaluations of a web-based system for training volunteer CHAs with little to no health background. Project HEAL aimed to assess whether web-based technology could be utilized in CHA training, with limited technical assistance, to implement an evidence-based cancer educational intervention in African American churches.

Evaluation of Project HEAL was based on the RE-AIM framework [1, 5, 6, 25]. We analyze the RE-AIM Framework outcomes in their order of occurrence: adoption, reach, implementation, efficacy, and sustainability. Due to the complexity of the intervention, multiple levels of analyses, and volume of data, this paper reports only on the first three of five main outcomes of Project HEAL at the organizational (church) and participants’ (CHA and workshop participant) levels: adoption, reach, and implementation. Efficacy and maintenance outcomes are discussed separately ﻿(Holt CL, Tagai EK, Santos SLZ, Scheirer MA, Bowie J, Haider M, Slade J, MQW: Is online comparable to in-person training for community health advisors conducting a church-based intervention?, submitted; Scheirer MA, Santos SLZ, Tagai EK, Bowie J, Slade J, Carter R, Holt CL: Dimensions of sustainability for a health communication intervention in African American churches: A multi-methods study, submitted). With regard to outcomes in the present analysis, we hypothesized that if the Traditional CHAs achieved greater adoption, reach, and implementation than the Technology CHAs, this would indicate the limitations of technology for training CHAs. However, if outcomes are comparable between the Traditional and Technology trained CHAs, this would have considerable positive implications for future dissemination/implementation and scale-up of EBIs delivered through CHAs, not only in faith-based settings but more broadly.

## Methods

### Participant recruitment

In Project HEAL, churches were recruited with the help of a community partner: a nonprofit organization with established relationships with many churches in the research target area. Churches were eligible if they had (a) a congregation size between 150 and 500, (b) at least 25 men and 25 women between the ages of 40 and 75, and (c) not hosted a breast, prostate, or colorectal cancer educational program in the past year.

After churches agreed to participate, a cluster randomized design [26] was used to randomly assign churches to one of two CHA training conditions: Traditional or Technology. For each condition, two CHAs (one male and one female) were recruited by their pastors to implement a series of three educational workshops that cover breast, prostate, and colorectal cancer education and screening. CHAs were eligible if they (a) regularly attended church services, (b) had regular access to the Internet and felt comfortable completing web-based training activities, (c) could complete Project HEAL CHA training, and (d) could lead the three-part workshop series.

Members of each church were recruited by the pastors and CHAs to participate in a CHA-led workshop series. Individuals were eligible to participate in the workshops if they (a) self-identified as African American; (b) were between the ages of 40 and 75; (c) had no personal history of breast, prostate, or colorectal cancer; (d) could attend the three-workshop series (or remaining workshops if enrolled after workshop 1); and (e) were able to complete project surveys.

Church pastors, CHAs, and participants completed informed consent at the time of Project HEAL enrollment. Churches received an incentive of $600 in two equal disbursements (at enrollment and after completion of the third workshop). CHAs received a total of $99 in two disbursements ($50 after CHA certification and $49 after completion of the workshop series). Participants received a $25 gift card for completing each of the baseline and 12-month follow-up surveys.

### Project HEAL intervention

In the “Traditional” (i.e., classroom, didactic) approach, CHAs were trained by study staff on 13 modules (e.g., overview of cancer, breast/prostate/colorectal cancer, how to conduct a workshop) over one 6-h or two 3-h sessions. Traditional CHAs received as much technical assistance/support from study staff as requested and became certified with a passing score (85% or greater) on an in-person knowledge examination. The second strategy, the “Technology” approach, covered the same material as the Traditional except that the CHAs completed their training and certification independently and according to their own schedule using a Web portal created by study staff. They received minimal technical assistance/support from study staff. After studying the web-based training videos that mirrored the same 13 modules covered in the Traditional approach, the CHAs took a web-based knowledge examination to become certified (with a passing score of 85% or greater). The CHAs in both approaches then delivered a series of three cancer early detection workshops in their churches covering breast, prostate, and colorectal cancer. Research staff attended each workshop to enroll participants, obtain informed consent, and observe and video record the presentations. The study protocol was approved by the University of Maryland, College Park Institutional Review Board. Additional information on Project HEAL intervention components are described in more detail elsewhere [24, 27].

### Measures

#### Adoption

Adoption is defined as the proportion of settings willing to initiate the intervention [5]. It was computed by dividing the number of churches that agreed to participate and initiated Project HEAL into the total number of churches that were approached for participation. The number of churches approached was tracked using a web-based tracking database accessible to study staff.

#### Reach

Reach is defined as the proportion of eligible individuals that participate in the intervention [5]. Reach was computed for each church by dividing the number of workshop participants enrolled in Project HEAL by the *estimated* number of eligible participants in that church, as provided by the pastor. The approximate number of eligible participants was obtained during a pastor-study staff interview that was conducted earlier in the project using our Faith-Based Organization Capacity Inventory (FBO-CI) instrument (described in ﻿Tagai EK, Holt, CL, Scheirer, MA, Santos, SLZ, Haider, M, Bowie, J, Slade, J, Whitehead, TL, Atkinson, M, & Wang, MQ: Assessing capacity of faith-based organizations for health promotion activities, submitted). The FBO-CI is a paper-and-pencil survey that assesses three structural areas of organization capacity specific to faith-based organizations: staffing and space, health promotion experience, and external collaboration. Pastors were asked the “estimated number of men/women age 40–75 who attend worship service weekly.”

#### Implementation

Implementation is defined as the fidelity of multiple intervention elements [5]. We further distilled the Project HEAL elements by evaluating adherence (consistency of delivery of core components as intended), dosage (frequency of use of core components), and quality (how closely the use of core components of the intervention resemble the theoretical ideal [28]. A variety of data sources were used to assess implementation: a research staff project tracking database, study staff-completed fidelity checklists based on workshop observations, CHA self-administered post-workshop surveys, and workshop participant post-workshop surveys.

*Adherence* was assessed at the CHA-level using four measures. (1) Spacing of workshops is the mean number of days between each workshop (i.e., days from workshop one to two and days from workshop two to three). Per the Project HEAL protocol, the suggested number of days between workshops was 30 days. This was decided upon so as to not over-burden the church calendar and participants’ schedules, not allow for too much time to pass between each workshop, and keep the project timeline moving. (2) Time to complete the workshop series was the mean number of days between workshop one and workshop three (expected number of days was 60). (3) CHA attendance was assessed for each church by summing each CHA’s attendance (1 = *attended* and 0 = *absent*) at all three workshop with scores ranging from 0 (no CHAs in attendance at any workshop) to 6 (100% attendance for both CHAs). (4) Workshop scheduling coordination assessed whether each workshop was scheduled in accordance to the weekly church calendar coded as 1 = *day with no other church events or conflicting events*, 2 = *workshop held in place of existing event* (e.g., bible study), and 3 = *workshop held directly after weekly service*. A total score was summed for each church across all three workshops (range = 3–9). Previous research has suggested that member participation is improved if health promotion workshops are held directly after a worship service or in place of a regularly scheduled event [29].

*Dosage* was assessed through four items: (1) number of workshops participants attended per church which ranged from 1 to 3; (2) percent of participants per church who enrolled at the first workshop, calculated by dividing the number of participants enrolled during workshop one by the total number of participants enrolled for each church; (3) participant exposure to educational print materials assessed using a single item on the participant 12-month follow-up survey that asked “how much of the booklets did you read?” Responses were recorded on a four-point Likert-type scale (1 = *none*, 2 = *some*, 3 = *most*, and 4 = *all*); and (4) coverage of Project HEAL PowerPoint slides by CHAs, assessed by study staff observation using a single item on the fidelity checklist for each workshop (1 = *none*, 2 = *partial*, and 3 = *full*) and was averaged across all workshops for each church (range = 1–3).

*Quality* was assessed through 7 items: (1) CHAs’ ratings of “I would recommend becoming a CHA to a peer”; (2) “I would recommend Project HEAL to men and women in my church” with a four-point Likert-type scale (1 = *strongly disagree* to 4 = *strongly agree*); (3) participant recruitment tools implemented by CHAs (e.g., called eligible participants, posted flyers, announcements during Sunday/Saturday service; max index = 39); (4) participant engagement in the workshops; (5) CHA engagement; and (6) CHA presentation competence assessed using a CHA fidelity checklist completed by research staff who observed each workshop. If a workshop was observed by more than one staff member, their observations were combined into a mean score for each workshop. All three measures were assessed using a four-point Likert-type scale: 1 = *poor*, 2 = *fair*, 3 = *good*, and 4 = *excellent*. CHA engagement and CHA presentation competence both had good internal reliability (*α* = .89 and *α* = .97, respectively).

#### Data analysis

All analyses were conducted using SPSS Version 23.0 (Armonk, NY). Descriptive statistics were calculated for all study variables. Bivariate analyses were completed to compare demographics for churches, pastors, CHAs, and participants in each study group. Though outcomes at the church and CHA level were limited by sample size and should be considered descriptive in nature, independent *t* tests were used to compare mean differences in participant-level outcomes, for which there was adequate sample size.

## Results

### Demographics

Church and pastor demographics (e.g., church building ownership, pastor education) were assessed using the FBO-CI that was completed shortly after the third Project HEAL workshop. Most churches were either Baptist or non-denominational (70.8%), and most pastors worked in their church full time (84.6%). CHA demographics (e.g., education, marital status, age) were assessed in the CHA certification training evaluation survey. A majority of the CHAs worked full- or part-time and over half (60.7%) had either their Associates or Bachelor’s degree. Participant demographics (e.g., age, education, employment status) were assessed on the Project HEAL baseline survey administered at enrollment. Participants had a mean age of 55.28 (*SD* = 9.28), indicated “some college” education (median), and had health insurance (93%; see Table 1).

**Table 1:** Project HEAL demographics (percentages shown unless otherwise indicated)

Church and pastor	Overall (*n* = 13)	Traditional (*n* = 7)	Technology (*n* = 6)
Church denomination			
Baptist	30.8	28.6	33.3
Non-denominational	46.2	42.9	50.0
Other	23.1	28.6	16.7
# of adult members (*M*, *SD*)	241.46 (178.87)	222.57 (165.41)	263.50 (207.04)
Pastor employment outside church			
Yes	15.4	14.3	16.7
No	84.6	85.7	83.3
Pastor education			
Some college	25.0	14.3^a^	33.3
Masters	41.7	42.9	33.3
Doctorate	33.3	28.6	33.3
Community health advisors	Overall (*n* = 28)	Traditional (*n* = 16)	Technology (*n* = 12)
Male	50.00	50.00	50.00
Female	50.00	50.00	50.00
Age (*M*,* SD*)	51.41 (12.84)	51.00 (11.84)	51.92 (14.51)
Education			
Less than HS diploma	3.6	0.0	8.3
HS diploma or GED	14.3	37.5	0.0
Some college	21.4	25.0	0.0
Associate’s degree	3.6	0.0	8.3
Bachelor’s degree	35.7	25.0	50.0
Master’s degree or higher	21.4	12.5	33.3
Marital status			
Single/never married	46.4	56.3	33.3
Married/living with partner	42.9	37.5	50.0
Separated/divorced	7.1	6.3	8.3
Widowed	3.6	0.0	8.3
Work status			
Retired	25.0	30.8	18.2
Disabled	4.2	7.7	0.0
Part-time	8.3	0.0	18.2
Full-time	62.5	61.5	63.6
Workshop participants	Overall (*n* = 375)	Traditional (*n* = 226)	Technology (*n* = 149)
Male	32.0	30.5	34.2
Female	68.0	69.5	65.8
Age (*M*, *SD*)	55.28 (9.28)	54.61 (9.14)	56.28 (9.42)
Education			
Less than HS diploma	6.0	5.4	6.8
HS diploma or GED	31.8	32.6	30.6
Some college	35.9	35.3	36.7
College graduate	26.4	26.7	25.9
Income (median)	$50–60 k	$50–60 k	$60–70 k
Marital status			
Single	28.34	31.36	23.81
Living with partner	1.09	1.36	0.68
Married	47.68	43.18	54.42
Separated/divorced	15.51	15.00	16.33
Widowed	7.36	9.09	4.76
Work status			
Retired	7.34	7.69	6.89
Disabled	19.84	18.10	22.45
Not currently working	11.96	13.12	10.20
Part - time	7.34	6.33	8.84
Full - time	53.53	54.75	51.70
Health insurance coverage	93.07	91.15	95.97

^a^ *n* = 6

### Adoption

**Fig. 1:**
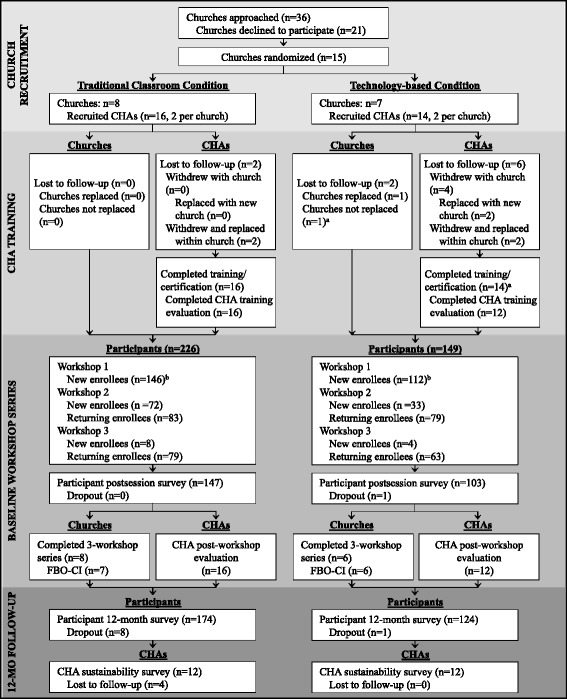
CONSORT flow diagram. *CHA* community health advisor, *FBO-CI* faith-based organizational capacity inventory. ^**a**^CHAs trained and certified, but church dropped out before first workshop date; church not replaced due to late drop out. ^**b**^Participants completed baseline survey upon enrollment in workshops 1–3

Adoption was assessed at the organizational (i.e., church) level and was 41% (see CONSORT flow diagram, Fig. 1). Thirty-nine churches were approached to enroll in Project HEAL. Of those, seven were ineligible due to size, 16 did not respond to our invitation to participate or gave varying reasons for declining participation, and 15 churches were included in the initial randomization process. One church dropped out shortly thereafter due to the dissolution of the church as an organization and was replaced. Another church dropped out later in the project timeline due to church relocation, dispersal of trained CHAs, and church organizational restructuring efforts but was not replaced because of late dropout. Adoption could not be compared by study group because adoption was assessed prior to randomization.

### Reach

Reach was assessed at the individual (i.e., workshop participant) level. Overall reach was estimated at 33.3% for the overall study and was marginally greater in the Traditional condition (43.2%) than in the Technology condition (21.7%; *p* < .10; see Table 2).

**Table 2:** Reach and implementation outcomes from Project HEAL churches

Outcome	Overall (*n* = 14^a^) *M* (*SD*)	Traditional (*n* = 8) *M* (*SD*)	Technology-based (*n* = 6) *M* (*SD*)	*p* value	Level of analysis
Reach					
% enrolled/eligible participants per church	33.31 (22.72)	43.23 (26.51)	21.74 (9.87)	.082	Participant
Implementation—adherence					
Spacing of WS in days (expected: ≤ 30 days)	38.00 (30.55)	42.38 (40.74)	32.17 (5.60)	–	Church
Time to complete WS series in days (expected: 60 days)	75.71 (60.89)	84.38 (81.21)	64.17 (11.21)	–	Church
CHA attendance per church (range = 0–6)	5.93 (0.27)	5.88 (0.35)	6.00 (0.00)	–	CHA
Workshop scheduling date (range = 3–9)	5.21 (2.69)	6.00 (2.78)	4.17 (2.40)	–	Church
Implementation—dosage					
# sessions attended (range = 1–3)	1.83 (0.28)	1.74 (0.17)	1.97 (0.34)	.127	Participant
% participants per church enrolled at WS 1 (range = 0–100)	70.84 (14.35)	66.22 (10.71)	77.00 (17.19)	.173	Participant
Educational booklets received (range = 1–4)	3.16 (0.88)	3.10 (0.90)	3.25 (0.86)	.250	Participant
All slides covered (range = 1–3)	2.86 (0.39)	2.92 (0.24)	2.79 (0.54)	–	CHA
Implementation—quality					
Recommend becoming a CHA (range = 1–4)	3.83 (0.39)	3.77 (0.44)	3.90 (0.32)	–	CHA
Recommend Project HEAL to men and women in my church (range = 1–4)	3.96 (0.21)	3.92 (0.28)	4.00 (0.00)	–	CHA
Participant recruitment by CHAs (max = 39)	19.40 (9.43)	19.13 (11.13)	19.80 (6.65)	–	CHA
Participant engagement (range = 1–4)	3.70 (0.22)	3.65 (0.25)	3.75 (0.19)	–	CHA
CHA engagement (range = 1–4)	3.53 (0.35)	3.50 (0.24)	3.57 (0.48)	–	CHA
CHA presentation competence (range = 1–4)	3.53 (0.35)	3.47 (0.37)	3.60 (0.33)	–	CHA

*Notes:* Statistical comparisons were only completed for participant-level data due to sample size

*CHA* community health advisor, *WS* workshop

^a^Due to church dropout, only 14 churches were included in analyses

### Implementation

*Adherence*, or the consistency of delivery of core components as intended, was assessed at the individual (i.e., CHA) level (see Table 2). Neither group of churches averaged completion of the three-workshop series in the target of 60 days. However, they did come close, with an overall average of 75.71 days (*SD* = 60.89). The Technology group came closer to the target (*M* = 64.17; *SD* = 11.21) than did the Traditional group (*M* = 84.38; *SD* = 81.21). There was considerable variability in the Traditional group with one church completing the entire series in 28 days and another church taking 283 days to complete all three workshops. With regard to spacing of the workshops, the churches were close to target overall (*M* = 38.00; *SD* = 30.55; expected *M* = 30 days), and the difference between study groups was negligible, again, with much greater variability in the Traditional group. CHA attendance at the workshops was very high (*M* = 5.93; *SD* = 0.27). In terms of scheduling a workshop date, workshops overall were scheduled in place of an already established event or after service (*M* = 5.21; *SD* = 2.69; range = 3–9).

*Dosage*, the frequency of use of core components, was assessed at the individual (e.g., workshop participant and CHA) level (see Table 2). Overall, participants attended an average of 1.83 of the three workshops (*SD* = 0.28); 70.84% (*SD* = 14.35) enrolled at workshop one. Participants reported reading a majority of the educational booklets (*M* = 3.16; *SD* = 0.88; range = 1–4). There was no statistically significant difference between study groups. Overall, CHAs covered a majority of the PowerPoint slides (*M* = 2.86, *SD* = 0.39; range = 1–3).

Lastly, *quality*, or how closely the use of core components of the intervention resembles the theoretical ideal, was assessed at both the workshop participant and CHA levels. Across both study groups, CHAs gave positive ratings of the intervention, reported using a mean of seven recruitment techniques (*SD* = 2.72; e.g., emails, calling participants, announcements during service) and ratings of participant engagement were uniformly high. CHAs in both groups delivered content with high levels of engagement (*M* = 3.53; *SD* = 0.35; range = 1–4) and confidence (*M* = 3.53; *SD* = 0.35; range = 1–4) (See Table 2).

## Discussion

Guided by the RE-AIM framework [5], we report on adoption, reach, and implementation outcomes at the organizational (church) and participant (CHA and workshop participant) levels from an implementation trial in which lay peer CHAs were trained using traditional classroom didactic methods compared with a new web-based system. Overall, we found adoption to be 41% at the church or organizational level. With regard to reach, the estimates suggest that overall, we were able to reach about one-third of eligible people in the churches with the Project HEAL intervention. The CHAs trained in person appeared to have greater reach than those trained using the web-based system. However, CHAs in the Technology group had slightly more “consistent” workshop participation among their church members (i.e., greater number of workshops attended), which likely has implications for some observed patterns in the efficacy data as reported by (Holt CL, Tagai EK, Santos SLZ, Scheirer MA, Bowie J, Haider M, Slade J, MQW: Is online comparable to in-person training for community health advisors conducting a church-based intervention?, submitted).

### Adoption

Although fewer than half of the churches approached for participation agreed to adopt Project HEAL, this rate is reasonable considering various factors: (1) Faith-based organizations are difficult to connect with and recruit, (2) there are historical issues of distrust of medical research and institutions [30, 31], and (3) the church’s mission is one of faith first and not health promotion [29]. Furthermore, there was no substantial monetary incentive for enrolled churches considering we asked for two committed volunteers to complete training and implementation as CHAs and for a 2.5-year overall church commitment to the project.

Adoption rates in the present study are lower than in several other church-based interventions. Heart, Body, and Soul, a smoking cessation intervention in African American churches had a 96% adoption rate [32], another church-based breast cancer screening intervention had a 92% rate [33], and a nutrition and physical activity intervention experienced 70% adoption among churches [34]. There are a number of factors that may influence adoption at the church level including recruitment techniques and the demands of the study. They may have been lower in the present case due to the 2.5-year commitment. Many church-based interventions were conducted over roughly a 1-year period [21–23, 32, 34], which may be perceived as more feasible for a church calendar to accommodate. Additionally, the intervention intensity of Project HEAL may have been difficult or intimidating for an individual with little to no health background. Some, but not all, churches provided reasons for not participating in Project HEAL including scheduling conflicts with the church calendar and proposed HEAL activities and pastor illness.

### Reach

Reach in Project HEAL was 33%, meaning that an estimated one third of eligible participants in each church enrolled in the intervention. Estimates of reach could be viewed as conservative, likely reflecting an underestimation, to the extent that pastors could have over-estimated the number of eligible individuals in their churches. We know from our related work in congregational assessment that it is often difficult to obtain accurate data on the number of church members, let alone the number of members in a particular age range and without a previous cancer diagnosis. This illustrates a challenge in obtaining accurate reach data for evaluative purposes, particularly in implementation research in community-based settings. However, any potential bias in this study would be expected to be equal across study groups due to the randomization.

Considering study group differences, reach was substantially greater in the Traditional churches than in the Technology group. However, the number of churches was small, limiting statistical power. Limitations in statistical power imposed by the clustered design are inherent in this type of research and hinder the ability to examine organizational level factors. Additionally, there may have been differences in how the CHAs engaged with the training that at present are unknowable in how they might have affected reach.

A potential method to increase reach is to widen the age eligibility for workshop participants in order to include younger congregants. Including younger people in cancer educational interventions has been recommended to us by community partners in several of our trials, in order to get people exposed to the information “early.” However, including young adults who do not fall within cancer screening guidelines does not provide a measurable behavioral outcome (e.g., cancer screening) for a trial, which from a research standpoint poses a considerable challenge. Future research and funding agencies should consider the benefit of including younger people in cancer educational interventions from a life course developmental perspective. Another benefit of widening the audience for these interventions is that most of the time people disseminate the information with others in their social networks. In the current case, 92% reported sharing the information with friends or family members ([Scheirer MA, Santos SLZ, Tagai EK, Bowie J, Slade J, Carter R, Holt CL: Dimensions of sustainability for a health communication intervention in African American churches: A multi-methods study, submitted).

### Implementation

This study illustrates the use of multiple measures for assessing implementation and suggests that no single measure adequately captures the many aspects of intervention delivery. Overall, both CHA training methods ultimately led to (1) the successful training and certification of lay CHAs and (2) the delivery of CHA-led cancer educational workshops for all churches. Most implementation measures showed consistently high levels of implementation suggesting that strong implementation of a complex intervention is in fact possible in a community setting. However, study staff did play a proactive role in communicating with CHAs to schedule workshops (to adhere to the project funding timeline) and were present at each workshop for evaluation purposes. Had Project HEAL been conducted using a more “real world” dissemination approach, implementation may have been more limited, thus illustrating an important gray area between “efficacy” and “effectiveness.” This is an area in which the field of translational research still has a considerable amount to learn, for example, how best to bring EBIs to bridge the gap between T3 to T4.

Ideally, the workshop series should have been conducted within 60 days (each workshop spaced about 1 month apart). Overall, it took about 76 days for churches to complete the workshop series, with the Traditional group taking an average of 84 days versus the Technology group with an average of 64 days. Within the Traditional group, one church took 283 days to complete the workshop series with much prompting from study staff to get the workshops completed. If study staff were not proactive in keeping this church active in the project, they would likely have been lost to follow-up. This again illustrates the somewhat unclear boundaries between “efficacy” and “effectiveness” along the translational continuum.

Participant attendance was only moderately high, with the average participant attending slightly fewer than two of the three sessions. This suggests that interventions conducted in churches may have difficulty obtaining high levels of consistency, if different people participate in different events. This finding is also consistent with the data about Project HEAL sustainability (Scheirer MA, Santos SLZ, Tagai EK, Bowie J, Slade J, Carter R, Holt CL: Dimensions of sustainability for a health communication intervention in African American churches: A multi-methods study, submitted), that the churches later conducted many different types of health promotion activities, but did not replicate the three-workshop cancer education series. Comparing the two groups, consistency was greater in the Technology group than in the Traditional group. It is possible that the CHAs in the Technology group had a more proactive approach in the techniques used (e.g., greater frequency of each technique, more frequent in-person contact) that resulted in greater workshop attendance among their members. Despite random assignment, CHAs in the Technology group may have developed a proactive approach to the project through the training process and the limited technical assistance provided from study staff, which may have fostered more active participant recruitment and workshop implementation.

### Strengths

There are a number of strengths to this study, including the implementation of an EBI and comparing a traditional classroom-based approach to training lay peer CHAs with a new web-based training program. Using a peer CHA approach combined with a web-based training modality designed to be accessible across various platforms may increase the ease of dissemination of other EBIs and to other contexts and populations provided that they can access the Internet. While the study was not an “effectiveness” trial, the findings help bridge the gap between efficacy and effectiveness, demonstrating Project HEAL’s implementation feasibility. Additionally, Project HEAL targets a population with disparate cancer outcomes in a limited resource environment.

### Limitations

The current findings must be interpreted in the context of several limitations. First, as previously mentioned, due to the clustered design, the study has limited statistical power when looking at the church/organizational level outcomes. Observed differences between study conditions for these outcomes may be viewed as trends or patterns, but they must be taken with caution and should be replicated. Findings may have limited generalizability with very small or very large churches as well as other geographical areas. As of 2015, Prince George’s County, MD, USA, had a population of about 900,000 with 11.7% being over 65 years of age and 64.6% identifying as African American. The county median household income was $73,856. In comparison, the USA overall had a population of 3.2 million with 14.9% being over 65 years of age and 13.3% identifying as African American. The national median household income was $53,882 [35]. Additionally, the web-based CHA training is limited to individuals with Internet access. Lastly, though we use multiple data sources, the implementation measures reflect process data specific to Project HEAL rather than established or validated instrumentation. This is often the case in evaluating intervention fidelity, though it makes for idiosyncratic assessment and difficult to compare across studies [36, 37].

## Conclusions

Project HEAL illustrated that use of a web-based portal for training lay, peer CHAs resulted in implementation outcomes comparable to use of a traditional classroom CHA training approach. CHAs trained with less staff contact and technical assistance were able to implement Project HEAL just as well as—or in some cases, marginally better than—CHAs trained with a traditional classroom hands-on approach. Furthermore, two companion papers to this study focused on participant outcomes and sustainability of Project HEAL, respectively. The former found that African American men and women who attended Project HEAL’s cancer education workshops given by CHAs trained using web-based methods learned just as much as those who attended workshops given by CHAs trained in the classroom (Holt CL, Tagai EK, Santos SLZ, Scheirer MA, Bowie J, Haider M, Slade J, MQW: Is online comparable to in-person training for community health advisors conducting a church-based intervention?, submitted). In the latter, (Scheirer MA, Santos SLZ, Tagai EK, Bowie J, Slade J, Carter R, Holt CL: Dimensions of sustainability for a health communication intervention in African American churches: A multi-methods study, submitted) indicates that substantial numbers of Project HEAL churches continued to provide health promotion activities after the initial three-workshop series. These findings imply strong potential for the field of implementation science to increase the reach of sustainable EBIs. Through the use of a web-based system to train lay, peer CHAs with limited technical assistance, there is high potential for successful implementation and sustainability of EBIs past the initial funding stage in which study staff support and funding typically cease to exist.

Future studies should conduct a cost/benefit analysis and examine the technical requirements needed to develop and maintain a web-based training model in order to determine the feasibility and efficiency of scale-up using this methodology. If the startup and maintenance costs of a web-based training portal are more cost-effective than a traditional classroom-based approach, it could have significant implications for the future of implementation research and the effort to eliminate health disparities. Finally, though Project HEAL and this series of analyses illustrated that web-based CHA training methods with some human contact and technical support result in strong implementation and reasonable participant level and sustainability outcomes, a broader scale-up and dissemination remains an elusive next step to reaching more people in more churches with evidence-based health information.

## References

[1] Glasgow RE, Marcus AC, Bull SS, Wilson KM (2004). Disseminating effective cancer screening interventions. Cancer.

[2] Fixsen DL, Naoom SF, Blase KA, Friedman RM, Wallace F (2005). Implement research: a synthesis of the literature.

[3] Proctor EK, Landsverk J, Aarons G, Chambers D, Glisson C, Mittman B (2009). Implementation research in mental health services: an emerging science with conceptual, methodological, and training challenges. Adm Policy Ment Health.

[4] Schoenwald SK, Hoagwood K (2001). Effectiveness, transportability, and dissemination of interventions: what matters when?. Psychiatr Serv.

[5] Glasgow RE, Vogt TM, Boles SM (1999). Evaluating the public health impact of health promotion interventions: the RE-AIM framework. Am J Public Health.

[6] Glasgow RE, Dzewaltowski DA, Estabrooks PA, Klesges LM, Bull SS (2002). Response from the Behavior Change Consortium Representatives and Translation Work Group: the issue is one of impact, not of world view or preferred approach. Health Educ Res.

[7] Chou WY, Prestin A, Lyons C, Wen KY (2013). Web 2.0 for health promotion: reviewing the current evidence. Am J Public Health.

[8] Griffiths F, Lindenmeyer A, Powell J, Lowe P, Thorogood M (2006). Why are health care interventions delivered over the internet? A systematic review of the published literature. J Med Internet Res.

[9] Eng TR (2002). eHealth research and evaluation: challenges and opportunities. J Health Commun.

[10] Fox S, Duggan M. Health Online 2013. Pew Research Center. 2013. http://www.pewinternet.org/2013/01/15/health-online-2013/. Accessed 08 Mar 2017.

[11] Strecher V (2007). Internet methods for delivering behavioral and health-related interventions (eHealth). Annu Rev Clin Psychol.

[12] Turner-Lee N, Smedley B, Miller J (2012). Minorities, mobile broadband and the management of chronic diseases.

[13] Ellis P, Robinson P, Ciliska D, Armour T, Raina P, Brouwers M, et al. Diffusion and Dissemination of Evidence-based Cancer Control Interventions. Agency for Healthcare Research and Quality (US), Rockville (MD). 2003. https://www.ncbi.nlm.nih.gov/books/NBK36992/. Accessed 08 Mar 2017.

[14] Witmer A, Seifer SD, Finocchio L, Leslie J, Oneil EH (1995). Community health workers: integral members of the health care work force. Am J Public Health.

[15] Community health advisors: Programs in the United States, health promotion and disease prevention. Volume II. Atlanta: U.S. Department of Health and Human Services, Public Health Service, Centers for Disease Control and Prevention, National Center for Chronic Disease Prevention and Health Promotion, Division of Chronic Disease Control and Community Intervention; 1994.

[16] Fendall R (1984). We expect too much from community health workers. World Health Forum.

[17] Giblin PT (1989). Effective utilization and evaluation of indigenous health care workers. Public Health Reports.

[18] Richter RW, Bengen B, Alsup PA, Bruun B, Kilcoyne MM, Challenor BD (1974). The community health worker. A resource for improved health care delivery. Am J Public Health.

[19] Walt GG, Lucy (1990). Community health workers in national programmes. Just another pair of hands?.

[20] Community health advisors: Models, research, and practice, selected annotations-United States. Volume I. Atlanta: U.S. Department of Health and Human Services, Public Health Service, Centers for Disease Control and Prevention, National Center for Chronic Disease Prevention and Health Promotion, Division of Chronic Disease Control and Community Intervention; 1994.

[21] Holt CL, Wynn TA, Litaker MS, Southward P, Jeames S, Schulz E (2009). A comparison of a spiritually based and non-spiritually based educational intervention for informed decision making for prostate cancer screening among church-attending African-American men. Urol Nurs.

[22] Holt CL, Litaker MS, Scarinci IC, Debnam KJ, McDavid C, McNeal SF, Eloubeidi MA, Crowther M, Bolland J, Martin MY (2013). Spiritually based intervention to increase colorectal cancer screening among African Americans: screening and theory-based outcomes from a randomized trial. Health Educ Behav.

[23] Holt CL, Klem PR (2005). As you go, spread the word: spiritually based breast cancer education for African American women. Gynecol Oncol.

[24] Holt CL, Tagai EK, Scheirer MA, Santos SL, Bowie J, Haider M, Slade JL, Wang MQ, Whitehead T (2014). Translating evidence-based interventions for implementation: experiences from Project HEAL in African American churches. Implement Sci.

[25] RE-AIM. http://www.re-aim.org/. Accessed 08 Mar 2017.

[26] Bland JM (2004). Cluster randomised trials in the medical literature: two bibliometric surveys. BMC Med Res Methodol.

[27] Santos SL, Tagai EK, Wang MQ, Scheirer MA, Slade JL, Holt CL (2014). Feasibility of a web-based training system for peer community health advisors in cancer early detection among African Americans. Am J Public Health.

[28] Century J, Cassata A, Rudnick M, Freeman C (2012). Measuring enactment of innovations and the factors that affect implementation and sustainability: moving toward common language and shared conceptual understanding. J Behav Health Serv Res.

[29] Campbell MK, Motsinger BM, Ingram A, Jewell D, Makarushka C, Beatty B, Dodds J, McClelland J, Demissie S, Demark-Wahnefried W (2000). The North Carolina Black Churches United for Better Health Project: intervention and process evaluation. Health Educ Behav.

[30] Ammerman A, Corbie-Smith G, St George DM, Washington C, Weathers B, Jackson-Christian B (2003). Research expectations among African American church leaders in the PRAISE! project: a randomized trial guided by community-based participatory research. Am J Public Health.

[31] Fowler BA (2006). Social processes used by African American women in making decisions about mammography screening. J Nurs Scholarsh.

[32] Voorhees CC, Stillman FA, Swank RT, Heagerty PJ, Levine DM, Becker DM (1996). Heart, body, and soul: impact of church-based smoking cessation interventions on readiness to quit. Prev Med.

[33] Sauaia A, Min SJ, Lack D, Apodaca C, Osuna D, Stowe A, MGinnis GF, Latts LM, Byers T (2007). Church-based breast cancer screening education: impact of two approaches on Latinas enrolled in public and private health insurance plans. Prev Chronic Dis.

[34] Winett RA, Anderson ES, Wojcik JR, Winett SG, Bowden T (2007). Guide to health: nutrition and physical activity outcomes of a group-randomized trial of an Internet-based intervention in churches. Ann Behav Med.

[35] United States Census Bureau. Quick Facts. https://www.census.gov/quickfacts/. Accessed 08 Mar 2017.

[36] Century J, Rudnick M, Freeman C (2010). A framework for measuring fidelity of implementation: a foundation for shared language and accumulation of knowledge. Am J Eval.

[37] Abry T, Hulleman CS, Rimm-Kaufman SE (2015). Using indices of fidelity to intervention core components to identify program active ingredients. Am J Eval.

